# Chronic *Cystoisospora belli* infection in a Colombian patient living with HIV and poor adherence to highly active antiretroviral therapy

**DOI:** 10.7705/biomedica.5932

**Published:** 2021-05-31

**Authors:** Ana Luz Galván-Díaz, Juan Carlos Alzate, Esteban Villegas, Sofía Giraldo, Jorge Botero, Gisela García-Montoya

**Affiliations:** 1 Grupo de Microbiología Ambiental, Escuela de Microbiología, Universidad de Antioquia, Medellín, Colombia Universidad de Antioquia Universidad de Antioquia Medellín Colombia; 2 Unidad de Micología Médica y Experimental, Corporación para Investigaciones Biológicas-Universidad de Santander, Medellín, Colombia Universidad de Santander Universidad de Santander Medellín Colombia; 3 Unidad de Investigación Clínica, Corporación para Investigaciones Biológicas, Medellín, Colombia Corporación para Investiga. Biológicas Corporación para Investigaciones Biológicas Medellín Colombia; 4 Grupo de Parasitología, Facultad de Medicina; Corporación Académica para el Estudio de las Patologías Tropicales, Universidad de Antioquia, Medellín, Colombia Universidad de Antioquia Universidad de Antioquia Medellín Colombia; 5 Centro Nacional de Secuenciación Genómica, Sede de Investigación Universitaria, Universidad de Antioquia, Medellín, Colombia Universidad de Antioquia Universidad de Antioquia Medellín Colombia

**Keywords:** Apicomplexa, diarrhea, HIV, acquired immunodeficiency syndrome, antiretroviral therapy, highly active, Colombia, Apicomplexa, diarrea, VIH, síndrome de inmunodeficiencia adquirida, terapia antirretroviral altamente activa, Colombia

## Abstract

*Cystoisospora belli* is an intestinal Apicomplexan parasite associated with diarrheal illness and disseminated infections in humans, mainly immunocompromised individuals such as those living with the human immunodeficiency virus (HIV) or acquired immunodeficiency syndrome (AIDS). An irregular administration of highly active antiretroviral therapy (HAART) in HIV patients may increase the risk of opportunistic infections like cystoisosporiasis. We describe here a case of *C. belli* infection in a Colombian HIV patient with chronic gastrointestinal syndrome and poor adherence to HAART. His clinical and parasitological cure was achieved with trimethoprim-sulfamethoxazole treatment. Although a reduction in the number of *C. belli* cases has been observed since the use of HAART, this parasite still has to be considered as a differential diagnosis of diarrheal disease in HIV/AIDS patients. Effective interventions enhancing adherence to HAART should be included in HIV patient care programs.

## Resumen

*Cystoisospora belli* es un parásito intestinal del filo Apicomplexa asociado con enfermedades diarreicas e infecciones diseminadas en humanos, principalmente en individuos inmunocomprometidos, como aquellos infectados con el virus de la inmunodeficiencia humana (HIV) o el síndrome de inmunodeficiencia adquirida (sida). El cumplimiento inadecuado de la terapia antirretroviral de gran actividad (TARGA) puede aumentar el riego de infecciones oportunistas, incluida la cistoisosporiasis. Se describe el caso de infección por *C. belli* en un paciente colombiano con HIV, que presentó un síndrome gastrointestinal crónico e incumplía el tratamiento con la TARGA. Después del diagnóstico parasitológico, el paciente fue tratado con trimetoprim-sulfametoxazol, lográndose la recuperación clínica y la cura parasitológica. Aunque se ha observado una reducción en el número de casos de *C. belli* desde la implementación de la TARGA, este parásito aún debe considerarse en el diagnóstico diferencial de las enfermedades diarreicas en pacientes con HIV/sida. Los programas de atención deben incluir intervenciones efectivas que potencien el cumplimiento de la TARGA en estos pacientes.

*Cystoisospora belli* is an obligate intracellular Apicomplexan parasite from the family Sarcocystidae responsible for human cistoisosporiasis, an intestinal cosmopolitan infection that is more frequent in tropical and subtropical regions ^1^^-^^3^. Although it was originally described in the genus *Isospora,* the identification of morphological, biological, and genetic differences between species from nonmammalian and mammalian hosts led to the creation of the genus *Cystoisospora*^4^, which includes mammalian species with facultative heteroxeny, oocysts containing two sporocysts with four sporozoites each, no Stieda bodies in sporocysts, and the ability to produce monozoic tissue cysts in intermediate or paratenic hosts ^1^^,^^5^^,^^6^.

*Cystoisospora belli* has a fecal-oral transmission cycle ^3^. Human hosts become infected through the ingestion of sporulated oocysts present in water or contaminated food. After consumption, the oocysts excyst in the small bowel and release sporozoites which invade enterocytes where the parasite multiplies asexually (endodyogeny) and sexually within the parasitophorous vacuole ^2^^,^^3^^,^^7^. *Cystoisospora belli* can also infect epithelial cells of bile ducts and gallbladder, especially in severely immunocompromised patients. Its life cycle is completed by the generation of an immature oocyst via fertilization of a female gamete (macrogamont) by a male gamete (microgamont) ^2^^,^^3^^,^^7^^,^^8^. Oocysts are excreted in stool and sporulate in the environment where they can survive for months.

*Cystoisospora belli* oocysts are generally bell-shaped, their size is 1737 x 8-21 um, and they have a very thin and smooth oocyst wall ^3^. Some sporozoites encyst in extra-intestinal organs (spleen, liver, mediastinal, trachea-bronchial, and mesenteric lymph nodes) and in intestinal lamina propria, particularly in immunosuppressed humans, producing monozoic tissue cysts ^2^^,^^3^^,^^7^^,^^9^. Until now, paratenic or intermediate hosts have not been identified in *C. belli's* life cycle.

Cystoisosporiasis is characterized by watery diarrhea, abdominal pain, fever, nausea, anorexia, significant weight loss, and may lead to dehydration and cachexia ^3^^,^^7^. Eosinophilia, steatorrhea, and the presence of Charcot-Leyden crystals are also common in *C. belli* infection ^3^. Symptoms are more severe in patients with immunity alterations, especially AIDS, but also in those with organ transplants subjected to immunosuppressive therapy, as well as patients with Hodgkin's disease, leukemia, and HTLV infection, among others ^2^^,^^3^^,^^7^. Immunocompetent hosts usually develop self-limited watery diarrhea and cases are mostly related to travelers from endemic areas.

Cystoisosporiasis therapy involves supportive treatment (fluid, electrolyte, and nutritional support) and antibiotics, trimethoprim-sulfamethoxazole (TMP-SMX) or cotrimoxazole, being the drugs of choice ^3^^,^^8^. Recurrence reports are common, which makes the infection difficult to eradicate and favoring its chronicity.

Despite its global distribution, *C. belli* is particularly endemic in tropical and subtropical areas from low-income countries in the Caribbean, Central and South America, Africa, and South-East Asia ^3^^,^^10^. Prevalence rates are significantly higher in HIV-infected patients compared to HIV-negative ones with values ranging between 0.4% and 28% ^3^. Data from endemic areas also vary among countries with prevalences ranging from 1.1% to 30% in South America ^3^. *Cystoisospora belli* epidemiology has been poorly evaluated in Colombia; the few studies conducted report prevalence rates between 0.06% and 8% in patients living with HIV and children ^11^^-^^15^^)^ (table 1). Here we describe a case of chronic diarrhea due to *C. belli* in a Colombian patient living with HIV and poor adherence to HAART.

**Table 1:** *Cystoisospora belli* prevalence data from studies in Colombia

Author	City	Population	Prevalence (%)	Reference
López, *et al.,* 1999	Bogotá	HIV patients	1.9	^11^
Arzuza, *et al.,* 2003	Cartagena	HIV patients	7.9	^12^
Botero, *et al.,* 2004	Medellín	HIV patients	1.9	^13^
Carmona, *et al.,* 2013	Turbo	Children	0.06	^14^
Lucero-Garzón, *et al.,* 2015	Florencia	Children	8	^15^

## Case report

We report a 57-year-old heterosexual man diagnosed with HIV nine years ago. He has two children and no stable partner and he currently attends a care program for people living with HIV. He lives in an urban area of Caucasia municipality in Antioquia, Colombia. The town is located 270 Km from Medellín, it is 150 meters above sea level, with an average temperature of 25°C to 32°C and a tropical monsoon climate. The patient works in different trades related to auto mechanics. He was admitted to the care program on July 3, 2018, and restarted the HIV second-line regimen of zidovudine/ lamivudine/lopinavir/ritonavir (AZT/3TC-LPV/r).

In the anthropometry evaluation, mild malnutrition (BMI=17.92) was reported but no other antecedents or comorbidities, except active smoking. He had received vaccination against hepatitis B, influenza, and pneumococcus a year earlier. Due to his history of poor adherence to antiretroviral therapy, follow-up was established to ensure treatment success.

He consulted with a four-month history of diarrhea, abdominal distension, flatulence, subjective weight loss, rectal bleeding, and hematochezia, but no fever. Gastrointestinal symptoms coincided with the start of the HAART. Initially, these symptoms were associated with the enhanced protease inhibitor included in the highly active antiretroviral therapy cocktail and, therefore, appropriate recommendations were given to improve gastrointestinal tolerance to these drugs.

**Figure 1:**
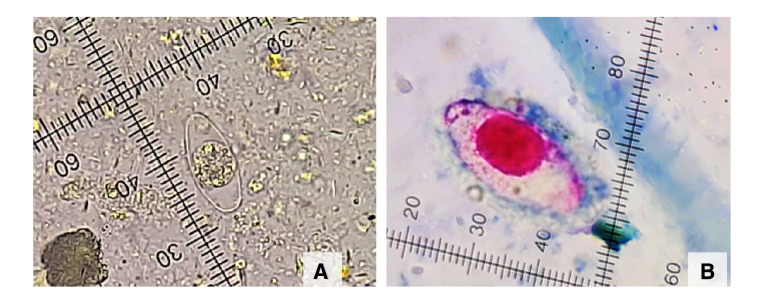
Inmature *Cystoisospora belli* oocyst with one sporoblast. **A.** Direct saline wet mount. 400X. **B.** Inmature *Cystoisospora belli* oocyst with one sporoblast. Kinyoun stain, 1.000X.

Besides, a parasitological study was requested to identify the infectious etiology of the clinical syndrome. *Cystoisospora belli* oocysts were detected by microscopic analysis of direct saline and iodine wet mounts of the patient's stool (figure 1A) along with the modified Ritchie sedimentation method. The diagnosis was confirmed by using the Kinyoun stain technique (figure 1B).

At the time of the diagnosis, the patient's HIV viral load was <40 copies/ ml and his CD4 T lymphocytes count was 465 cells/mm^3^. The hematological parameters were normal and he was negative for hepatitis C (antibodies) and hepatitis B (antigens and antibodies).

The patient was prescribed trimethoprim/sulfamethoxazole (TMP/SMX) DS 160/800 mg every 12 hours for two weeks and then every 24 hours for the same period. A full recovery of the gastrointestinal symptoms was achieved and the anthropometric data of the patient indicated a eutrophic nutritional status (BMI=19.21) and a weight gain of 6 kg since the last control.

### Ethical considerations

The study protocol followed the ethical guidelines of the 2013 Declaration of Helsinki. All approvals observed the ethical standards set by the Ministry of Health of Colombia (Resolution 008430 of 1993). The patient signed an informed consent explaining the purposes, benefits, and risks of the study. The patient's data were kept in confidentiality. The *Universidad de Antioquia* Bioethics Committee approved the investigation protocol (official document N° 17-06-760).

## Discussion

Diarrhea is a frequent and often inadequately treated complication in people living with HIV, which contributes to the reduced life quality and survival of these patients. It is estimated that 50% - 60% of AIDS patients have diarrhea at some point during their illness ^16^ and nearly 90% of them in developing countries ^17^. Hence, the etiology of this symptom should be carefully studied to reduce the morbidity and mortality associated.

Although HAART has significantly reduced the incidence of most opportunistic infections, parasite-related diarrhea remains a common problem in patients living with HIV, particularly in developing countries where they are probably underestimated ^10^^,^^18^. *Cystoisospora belli* is one of the parasites most frequently associated with diarrhea in patients living with HIV. During the 90s, *C. belli* cases were described worldwide, however, in the HAART era, cystoisosporiasis cases have concentrated in tropical and subtropical areas of developing countries ^10^. A recent global systematic review and meta-analysis of parasite prevalence in patients living with HIV showed a heavy burden of *Cryptosporidium,* microsporidia, and *Cystoisospora* infection, especially in low-income countries and sub-Saharan Africa ^10^. The few studies found in Latin American countries indicate that the epidemiology of this parasite is poorly known in our continent.

Here, we present a *C. belli* infection case associated with chronic diarrhea in a Colombian patient from Caucasia municipality where tropical monsoon climate facilitates the sporulation of the parasite oocysts and their transmission to humans. Additional factors are probably related to an increased risk of *C. belli* infection including the lack of access to sanitation facilities and safe water usually present in this region of the country. Sanitation conditions are determinant factors for cystoisosporiasis incidence and permanence.

The patient's poor adherence to HAART is an issue of concern. Irregular administration of HAART in patients living with HIV not only reduces the likelihood of optimal viral suppression but also increases the risk of opportunistic infections such as cystoisosporiasis. Therefore, adherence to treatment has become crucial in the clinical and public health management of HIV infection ^19^. Unfortunately, adverse drug reactions are one of the main causes for HAART discontinuation; several adverse effects related to the HAART drug cocktail components are known, among them protease inhibitors ^19^. Initially, gastrointestinal symptoms in our patient were thought to be related to the HAART therapy, but parasitological analysis confirmed the infectious etiology of the syndrome detecting *C. belli oocysts* in his stool.

*Cystoisospora belli* chronicity could be related to infection persistence possibly associated with the reactivation of the tissue cysts formed by the parasite in extra-intestinal organs or in the lamina propria of the intestine ^3^. Cystoisosporiasis recurrences are increasingly observed in patients living with HIV/AIDS, particularly in those who do not respond properly to the treatment ^20^. The standard treatment for *C. belli* infection is oral trimethoprim-sulfamethoxazole for 7 to 10 days in immunocompetent patients; however, immunosuppressed patients usually need longer treatment schedules or high doses, as well as maintenance therapy with cotrimoxazole to prevent relapses, which occur in nearly 50% of patients following discontinuation of initial therapy ^21^.

In this case, the patient recovered from the symptoms and responded well to the treatment administered after the parasitological diagnosis. Persistent infection was most likely due to the absence of an effective treatment given the late detection of *C. belli.* One of the major problems associated with the diagnosis of intestinal parasites, including *C. belli,* is the intermittent shedding of their oocyst in the feces favoring false-negative results. Generally, multiple samples collected on non-consecutive days improve the sensitivity of the direct parasitological analysis ^21^.

## Conclusion

Although cystoisosporiasis is considered a rare condition, it should be considered in the differential diagnosis of chronic diarrhea in immunosuppressed individuals, especially HIV/AIDS patients from tropical and subtropical regions. This report addresses the importance of a correct and timely parasite diagnosis to improve patients' outcomes. Laboratory technicians must be trained on parasite identification and the factors that improve the sensitivity of diagnostic techniques. Finally, the availability of parasite DNA will allow us to carry out future molecular studies regarding species confirmation and parasite's genetic diversity in our population thus contributing to the knowledge of its biology.
